# Screening for Food Insecurity in Six Veterans Administration Clinics for the Homeless, June–December 2015

**DOI:** 10.5888/pcd14.160375

**Published:** 2017-01-12

**Authors:** Thomas P. O’Toole, Christopher B. Roberts, Erin E. Johnson

**Affiliations:** 1 National Center on Homelessness Among Veterans, Providence, Rhode Island; 2 VA Center for Health Equity Research and Policy, Providence, Rhode Island; 3 Providence VA Medical Center, Providence, Rhode Island; 4 Alpert Medical School at Brown University, Providence, Rhode Island.

## Abstract

**Objective:**

We assessed findings from a food-insecurity screening of a national sample of Veterans Administration clinics for homeless and formerly homeless veterans.

**Methods:**

We reviewed results from initial screenings administered at 6 Veterans Administration primary care clinics for the homeless and responses from clinic staff members interviewed about the screening program.

**Results:**

A total of 270 patients were screened. The average age was 53 years, and most were male (93.1%). Screening showed a high prevalence of food insecurity. Of the 270, 48.5% reported they experienced food insecurity in the previous 3 months, 55.0% reported averaging 2 meals a day, and 27.3% averaged 1 meal a day. Eighty-seven percent prepared their own meals, relying on food they bought (54.2%), help from friends and family (19.1%), and soup kitchens and food pantries (22%); 47.3% received Supplemental Nutrition Assistance Program benefits (food stamps). Additionally, of those who screened positive for food insecurity 19.8% had diabetes or prediabetes, and 43.5% reported hypoglycemia symptoms when without food. Clinic staff members responded positively to the screening program and described it as a good rapport builder with patients.

**Conclusions:**

Integrating screening for food insecurity among patients in clinical settings was well received by both patients and health care providers. Addressing these positive findings of food insecurity requires a multidisciplinary health care approach.

## Introduction

Food insecurity is a serious and significant issue affecting many Americans either daily or at the end of the month when financial resources are exhausted (1,2). Food insecurity is a measure of how well our social safety net is working. In the United States: 42.2 million people were in a low food secure household (reduced quality, variety, or desirability of diet without reduced food intake) in 2015, and 10.9 million adults lived in very low food-secure households (disrupted eating patterns and reduced food intake) (3), including 27.0% of Iraq–Afghanistan war veterans (4).

Research shows that the issue of food insecurity is particularly relevant for those who are homeless or housing insecure (5). In one study, 76.7% of respondents experiencing housing insecurity were also experiencing food insecurity, which was significantly associated with difficulty accessing health care and with relying on the emergency department for their health care needs (6). The risk of food insecurity also extends to those who recently moved into independent housing where they may not be accustomed to purchasing and preparing foods, where they may be geographically isolated from grocery stores and food outlets, or where their resources for purchasing food may be limited.

The consequences of food insecurity are significant and potentially life-threatening. One study documented the inherent risks from low blood sugar among elderly people with diabetes who have other significant illnesses and don’t have a stable source of food (7,8). Specific medications beyond long-acting sulfonylureas (eg, β blockers, trimethoprim-sulfa, and haloperidol) are linked to hypoglycemia among the food insecure, (9). Hypoglycemia, cognitive dysfunction, and an increased risk of falls are just some of the complications and consequences of food insecurity. Another study found that risk for hospital admissions for hypoglycemia increased 27% in the last week of the month among low-income populations, typically when food stamps and supplies at food pantries ran low or were exhausted (10,11).

Our understanding of the role that primary care clinics can play in screening for food insecurity and addressing its clinical consequences and social drivers is limited. Integrating public health practice into clinic settings by incorporating social determinants of health in screening and interventions related to broad needs, such as housing insecurity and food insecurity, is an important opportunity that can improve health (12,13). In this article, we describe a clinic-based food insecurity screening program that we pilot-tested in 6 geographically and demographically distinct (East Coast, West Coast, Midwest, urban, rural) Veterans Affairs (VA) clinics caring for veterans who were homeless, at-risk for homelessness, or recently homeless living in transitional or supportive housing. We found a high prevalence of food insecurity among patients in this clinic setting and several implementation challenges and opportunities reported by front-line health care providers.

## Screening for Food Insecurity

From June through December 2015, we screened new patients who enrolled in care at one of 6 VA Homeless Patient Aligned Care Teams (H-PACTs) (12). The screening instrument was adapted from a one-question query (“In the past month, were there times when the food for you just did not last and there was no money to buy more” [14]) that was validated in a neighborhood health center serving a low-income urban community. We modified that single question to ask about food insecurity in the past 3 months to capture more information on its prevalence. Positive responses were then followed by 6 additional questions asking 1) where patients obtained their food (“Where do you get your food from?” with nonexclusive options of soup kitchens, local markets, shelter, family/friends), 2) the number of meals they ate per day (with options of 1, 2, or 3 meals/day), 3) whether they prepared their own meals (answering yes or no to the question, “Do you prepare your own meals?”), 4) whether they received food stamps (answering yes or no to the question, “Do you receive food stamp/SNAP assistance?”), 5) whether they had diabetes (answering yes or no to the question, “Do you have diabetes or trouble controlling your blood sugar level?”), and 6) whether they experienced hypoglycemia symptoms (answering yes or no to the question, “When you have not had enough food, have you ever noticed symptoms like feeling anxious, sweaty, feeling like you were going to pass out, chest pain?”) . Responses were then correlated with information from the patient’s electronic medical record on active diagnoses and currently prescribed medications associated with hypoglycemia. The questions were part of a templated note within the electronic medical record and were designed to be administered by anyone on the health care team with the patient referred to the nurse, primary care provider, social worker, or nutritionist depending on identified needs. A validated single-question screening instrument was chosen over other multistep or more detailed assessments of food insecurity (15) to minimize potential respondent or interviewer burden and with the expectation that the single question could be readily incorporated into other screenings that were already part of a clinic visit. The screening question and follow-up queries took 3 to 5 minutes to administer and record.

For analyses of the pilot responses, cohort and associated clinical reminder data were created from Structured Query Language extracts of the Health Factors table from the VA Corporate Data Warehouse (CDW) (16) and coupled with relevant patient demographic characteristics, information from the clinic visit, and additional diagnoses from CDW data fields. SAS 9.4 (SAS Institute Inc) was used to clean, format, and analyze data. We also surveyed team members responsible for championing the pilot program at each of the participating sites (3 nurses or nurse practitioners, 2 social workers, and 1 primary care provider). We used theme-based, open-ended questions for a group interview with representatives from all 6 pilot sites and semistructured follow-up interviews with each clinic’s teams. Questions focused on patient and staff reactions to the screening questions, perceived understandability and acceptability of the screening, operational challenges to administering the questions, and issues that arose in responding to positive screens (ie, indication of food insecurity).

## Food Insecurity and Associated Problems

A total of 270 patients were screened. Data were not available on those who refused the questionnaire or were not screened for other reasons. Participants were screened only once. The average age was 53 years (range: 23–85 y), and respondents were predominantly men (93.1%). Overall, 48.5% reported food insecurity during the previous 3 months. Among those reporting food insecurity, 87.0% prepared their own meals, relying on food they purchased (54.2%), soup kitchens and food pantries (22.9%), shelters (14.5%), and help from friends or family (19.1%); 47.3% were receiving food stamps, 62.6% were in their own apartment, and 26% were in a transitional housing program where they were responsible for some of their meals. Most (55.0%) reported having 2 meals per day, and 27.3% reported only one meal per day. Overall, 22.1% had depression, 22.0% had psychoses, 25.2% abused alcohol, and 19.8% had diabetes or prediabetes; 43.5% reported that they experienced hypoglycemia symptoms (eg, anxiety, sweating, chest pain) when without food. The Figure describes the screening process, how health care providers addressed patients who were food insecure, and data collection and follow-up.

**Figure:**
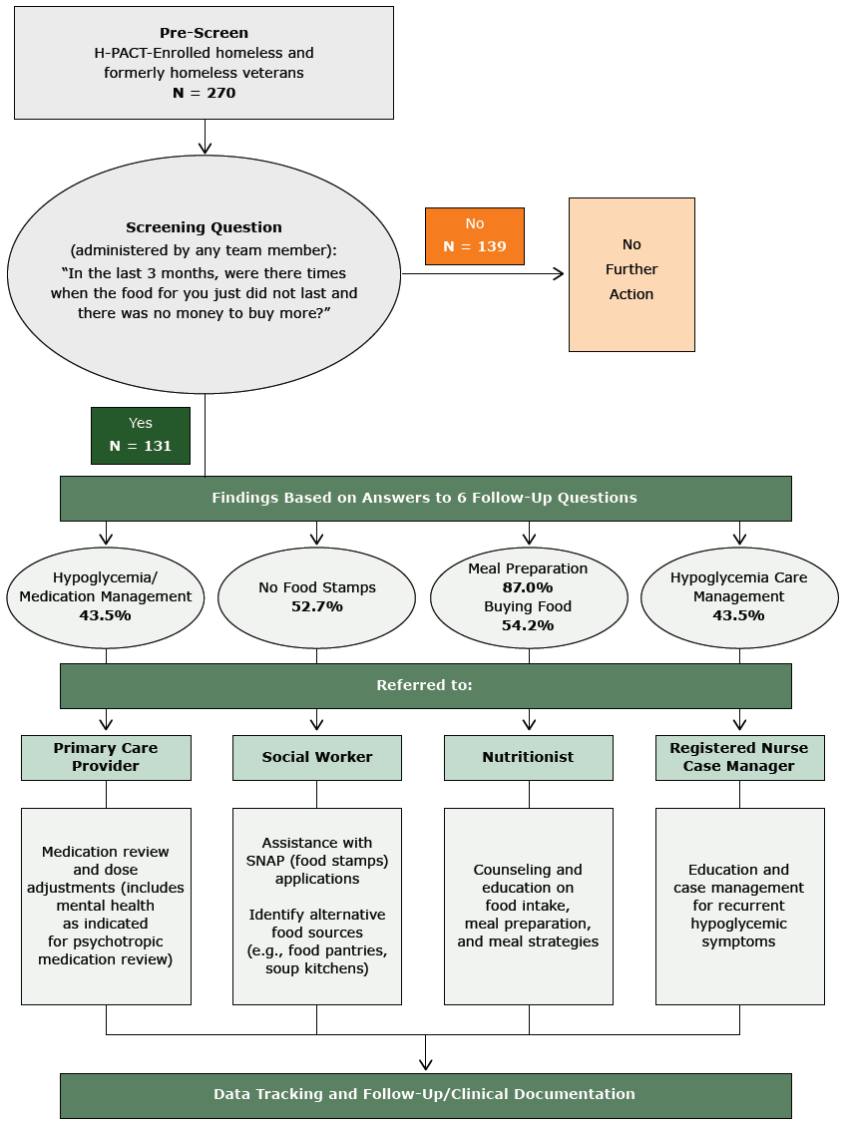
Patient flow and screening results of screening program for food insecurity conducted in 6 Veterans Administration clinics, June–December 2015.

When health care providers at pilot clinics were queried on user acceptance and implementation issues related to the screening, all universally endorsed the program. In the group interview of health care teams at the 6 pilot sites, staff members from 2 sites noted favorable feedback from patients, and one noted that the screening was a good rapport builder, that patients appreciated being asked these questions, and that the screening helped patients build a stronger connection with the health care team. No team found the questions burdensome. Four teams said the follow-up questions highlighted the complexity of issues underlying food insecurity and the need for a well-integrated, multidisciplinary approach to food insecurity. Examples of remarks were “This is more than a nursing or social work issue,” and “They may have food stamps but that doesn’t mean they are making good decisions or know how to make them [the food stamps] last.” Participants also cited a need for cross-training on issues and resources related to food insecurity for all team members. Teams also identified challenges with real-time triage and referral to extended-team members. Most teams reported using a co-signed electronic note or consult referral as a back-up when a real-time referral was not possible.

## Public Health Implications

We offer 3 observations from our pilot test of screening for food insecurity in a high-risk, vulnerable population in primary care practices. First, incorporating a food insecurity screening in a primary care setting was well-received by both providers and patients. Second, not only was the prevalence of food insecurity high (almost 50%) in the population studied, but several presumptions were also challenged: a) the receipt of food stamps was not necessarily protective from food insecurity for all recipients and b) although use of community resources (food pantries and soup kitchens) was extensive, most respondents also purchased some of their own food, suggesting a need for an intervention that focuses on how to extend a food budget. The rate of self-reported hypoglycemia was higher than what would be expected from diabetes alone, suggesting contributing effects of other medications or factors such as alcohol abuse. This finding merits further investigation. Finally, asking only about food insecurity was not enough. The additional context provided by the follow-up questions and breadth of different responses underscored that the needs of these patients extend beyond those available from one health care provider or one health care discipline. These findings show how addressing issues related to the social determinants of health can be incorporated into primary care settings, especially those that care for populations for whom both housing insecurity and food insecurity are active social mediators of health (13).

Our study has limitations. Screening was limited to homeless and formerly-homeless veterans cared for within the VA in a primary care setting tailored to the homeless. Our experience may not necessarily be generalizable to other populations or settings. More work is needed to assess the viability and appropriateness of the follow-up questions and triaging of patients with positive screening results among health care team members. Finally, our study addressed only the implementation of the screening and did not assess the clinical team’s response to a positive result. Further evaluation is needed to determine what, if any, effect this may have on addressing food insecurity within this population and whether how the screener administers the questionnaire has any clinical or social impact.

Prevalence of food insecurity is high among patients in clinics that serve homeless populations. Screening for food insecurity in these clinical settings offers an opportunity to integrate public health practice into clinical care and to incorporate social determinants of health into clinical practice.
